# Grynfeltt Hernia (GH): A Rare Case of Hernia

**DOI:** 10.7759/cureus.42478

**Published:** 2023-07-26

**Authors:** Vasco S Cardoso, Bernardo P Vasconcelos, Carlos Ascensão

**Affiliations:** 1 General Surgery, Hospital De São Francisco Xavier, Centro Hospitalar Lisboa Ocidental, Lisbon, PRT; 2 General Surgery, Lusíadas Hospital, Lisbon, PRT; 3 General Surgery, Hospital São Francisco Xavier, Centro Hospitalar Lisboa Ocidental, Lisbon, PRT

**Keywords:** case report, sandwich technique, superior lumbar triangle, grynfeltt hernia, lumbar hernia

## Abstract

Grynfeltt hernia (GH) is extremely rare among all abdominal wall hernias, so both diagnosis and treatment can be challenging. Surgery, open or laparoscopic, is the only definitive treatment. We present a case of a 71-year-old woman with GH (initially misdiagnosed as a lipoma), its approach, and treatment. We performed a hernioplasty with two meshes (preperitoneal and subaponeurotic position) by an open approach: *Sandwich* technique. This technique is safe, feasible, and associated with no short-term complications or relapses.

## Introduction

The anatomical area delimited superiorly by the 12th rib, inferiorly by the iliac crest, medially by the erector spinae muscles, and laterally by the external oblique muscle is called the lumbar region [1]. Here, two weak triangular-shaped abdominal wall areas can be identified: the inferior lumbar triangle, described by Petit in 1783, delimited by the iliac crest inferiorly, the latissimus dorsi muscle medially and the external oblique muscle laterally and the superior lumbar triangle (Grynfeltt's triangle), described by Lesshaft (1870) and Grynfeltt (1886), shaped like an inverted triangle (with the apex directed inferiorly) and delimited medially by the erector spinae muscle, laterally by the internal oblique muscle and superiorly by the 12th rib [1-2].

Although it was initially mentioned by Barbette in 1672 [2], the first reported case of lumbar hernia (LH) was reported by Garangoet in 1731 [3] and, up to date, there are approximately 300 case reports published [2-3]. LH is extremely rare. They are more common in men, aged 50-70 years, usually unilateral on the left side, and are caused by a rupture in the transversal fascia in correspondence to the Grynfeltt space [2-3]. Among all the abdominal wall hernias, the prevalence of Grynfeltt hernia (GH) is only 2%: 20% of them are congenital (due to embryogenetic defects such as anomalous vertebral and rib defects) and 80% are acquired; 55% of the acquired ones are either spontaneous or primary (atraumatic) and the remaining 25% are secondary, caused by injury or surgery [2-3].

The clinical manifestation of this herniation is extremely rare [3]. LH may be associated with nonspecific abdominal disturbances (nausea, vomiting, or pain), urinary problems, low back pain, and swelling which increases with cough in the lumbar region [1, 4]. Bowel obstruction due to incarceration can occur in 9%-25% of cases and strangulation in 8%-10%, so prompt treatment of LHs is essential [3-4].

Diagnosis of an LH is clinical, with evidence at palpation of swelling of the lumbar area characterized by volume increase during a Valsalva maneuver [1]. Differential diagnosis must be made with a lipoma (the most published misdiagnosis [3]), fibroma, hematoma, abscess, kidney tumors, muscle hernia, panniculitis, pannicular lumbosacroiliac hernia, and rarely with sarcoma [1-2, 5-6]. An ultrasound scan (US) is the first exam if any clinical suspicion arises [4]. However, the US does not adequately demonstrate the contents and their relation with the abdominal wall [3-4]. So, confirmation of these conditions frequently requires a CT scan or MRI, CT scan being the study of choice [5]. These methods are useful to better assess the muscular walls, the anatomical relationships, and the size of the hernia and its contents allowing the surgeon to plan the surgery [3-4]. Surgery is the only definitive treatment option and it can be by open or laparoscopic approach. We present a case of GH treated by open surgery in our institution. 

## Case presentation

**Figure 1:**
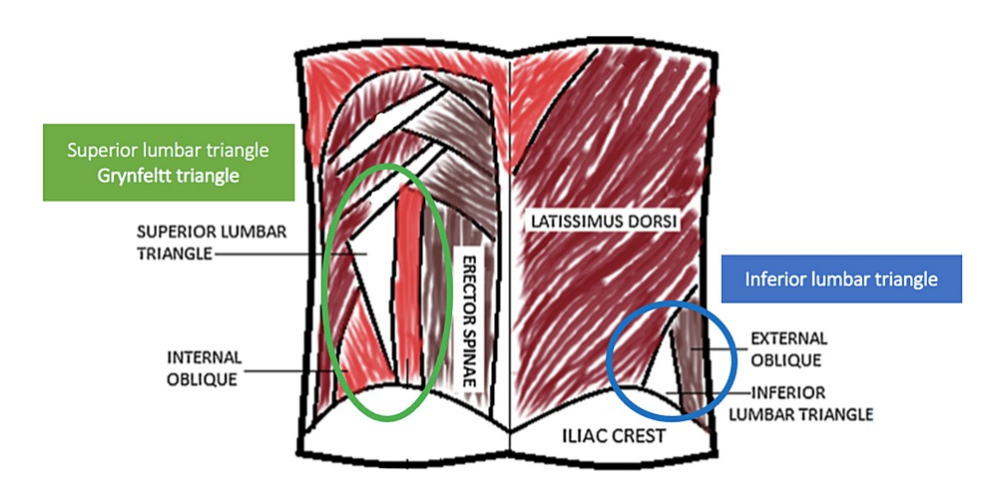
Diagrammatic representation of lumbar triangle anatomy. Image adapted from Sundaramurthy et al. [2]

Please see below Figure 1.

**Figure 2:**
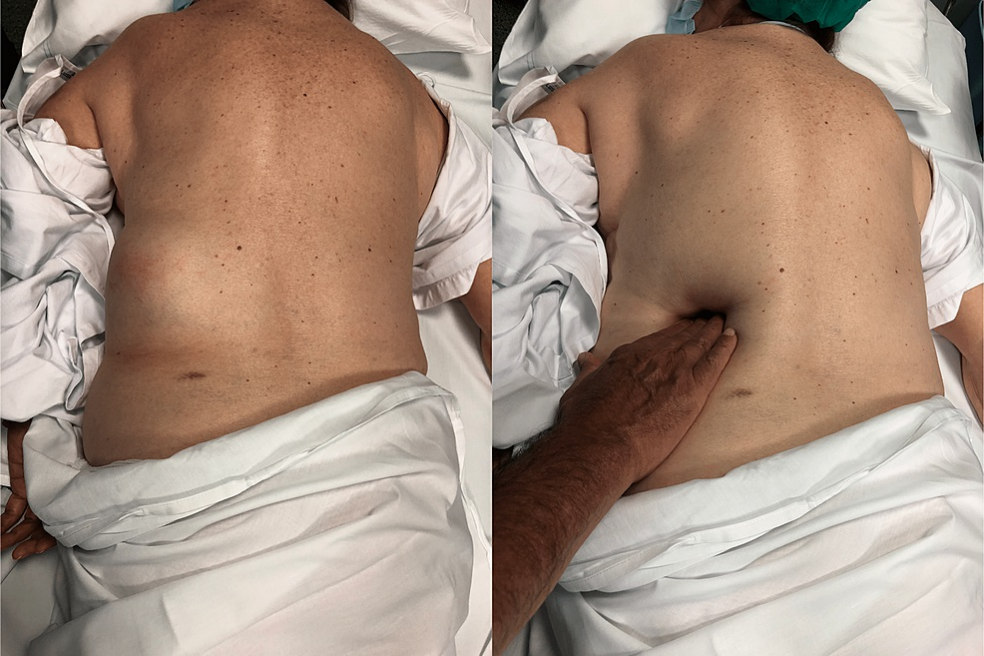
Left - Objective examination of soft swelling on the left lumbar region. Right - Manual reduction of the suspected GH. GH, Grynfeltt hernia

We present the case of a 71-year-old retired woman, with 69 kg and 1.56 m (body mass index, BMI - 28.4 kg/m2). Her past medical history is gastroesophageal reflux disease and her surgical history includes excision of squamous cell carcinoma on her right leg, right shoulder arthroplasty, total hysterectomy, appendicectomy, and orthopedic surgery because of proximal humerus fracture. She was medicated with omeprazole, naproxen, calcium carbonate, glucosamine, simethicone, and ginkgo biloba. In a follow-up appointment after the orthopedic surgery, she spontaneously reported swelling on the left flank, with about 1 year of evolution and progressive growth. She reported pain in that location and difficulty lying down, causing occasional discomfort. She denied a history of heavy lifting or trauma. On examination, she presented a soft swelling on the left lumbar region, bordered superiorly by the 12th rib, medially by the erector spinae muscle, and laterally by the internal oblique muscle (Figure 2).

**Figure 3:**
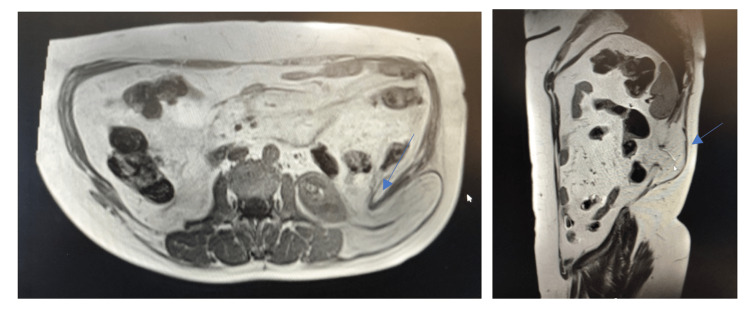
MRI with blue arrows showing herniation through the superior lumbar triangle.

A soft tissue ultrasound was requested and described "space-occupying, gross, oval, hypoechogenic lesion, measuring 12 cm x 3 cm x 11 cm suggesting lipoma.” Due to the uncertainty of the diagnosis, an MRI was requested and showed aspects compatible with GH: “hernia sac measuring 10.5 cm x 3.6 cm x 9.7 cm, containing fat and retroperitoneal vessels and hernia neck measuring 3.4 cm” (Figure 3).

**Figure 4:**
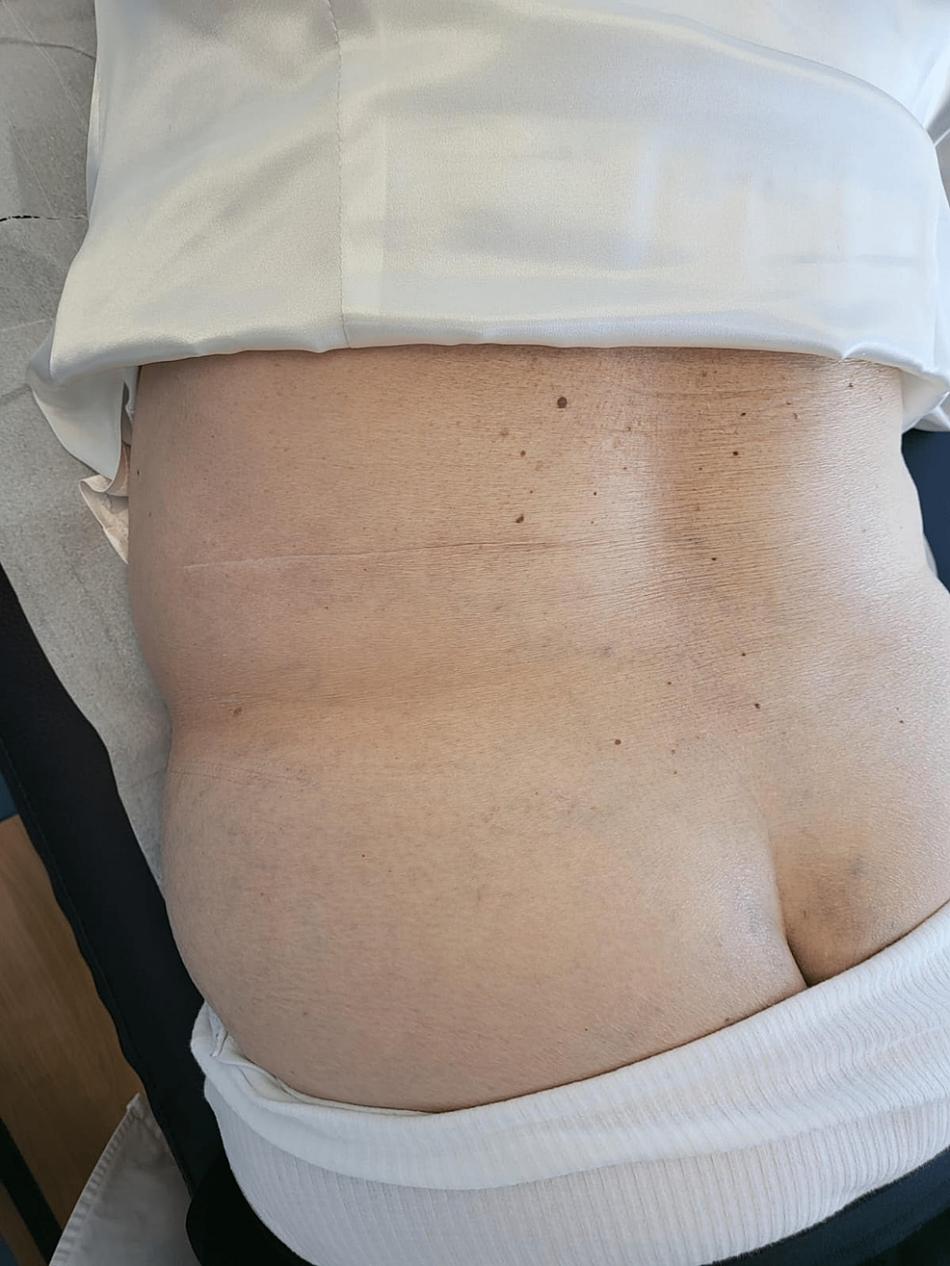
Fourth-month follow-up examination picture showing the scar and the GH corrected. GH, Grynfeltt hernia

The patient was observed on General Surgery consultation and was scheduled for an elective open repair. Under general anesthesia and open approach, she underwent surgical correction of the defect with placement of preperitoneal Ventralex® (BDI Surgery, Rhode Island) and subaponeurotic polypropylene meshes, separated by a muscular layer -- “Sandwich technique” (see Technical Report). The postoperative course was uneventful. She was discharged on the second postoperative day. At follow-up fourth month, she denied any complaints and no relapse was seen on examination (Figure 4).

Technical Report

**Figure 5:**
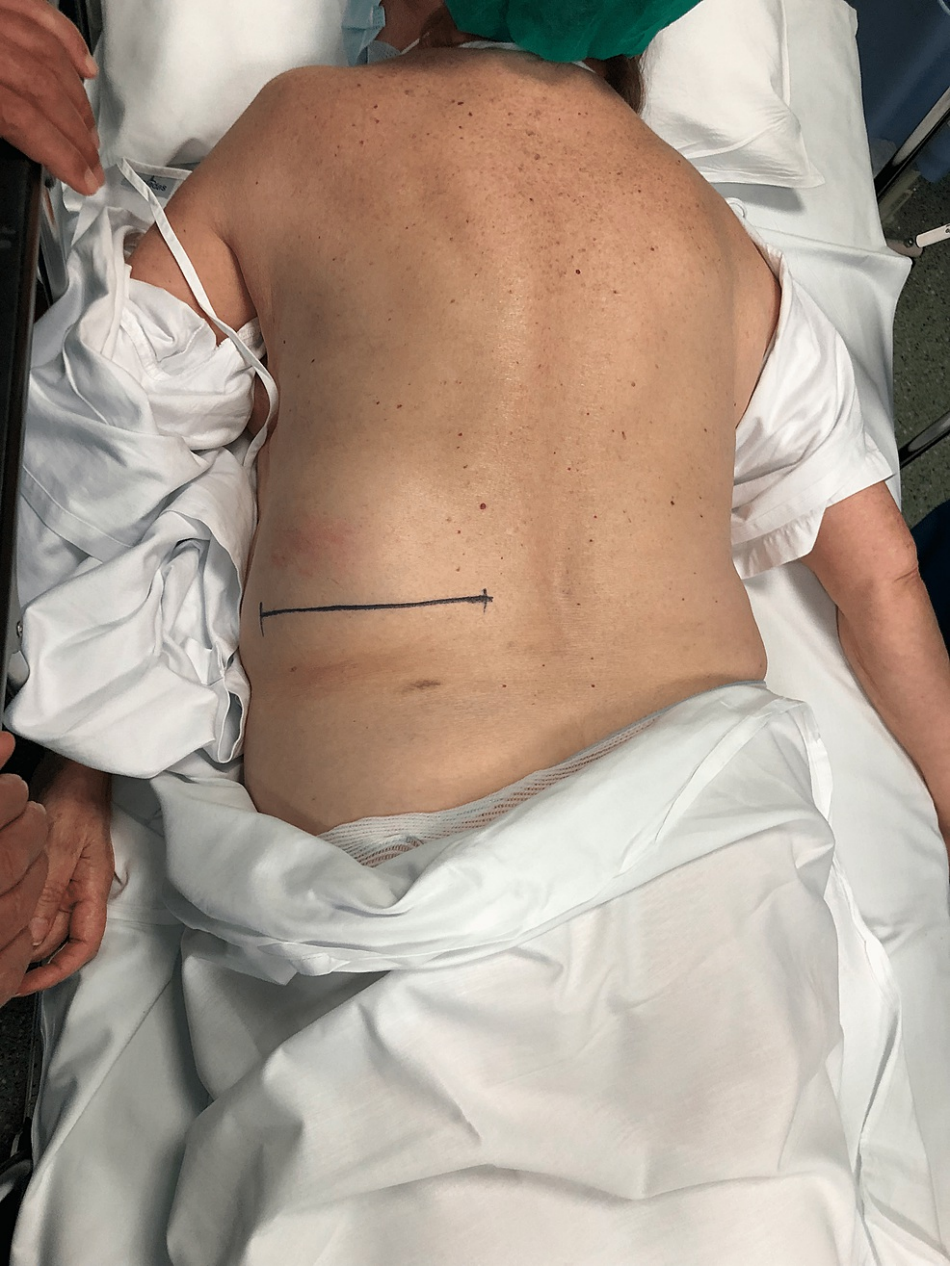
Local of the incision of left lumbotomy.

The procedure was carried out with the patient in the ventral decubitus. A left lombar region incision (around 7 cm) was performed (Figure 5), followed by plane dissection. 

**Figure 6:**
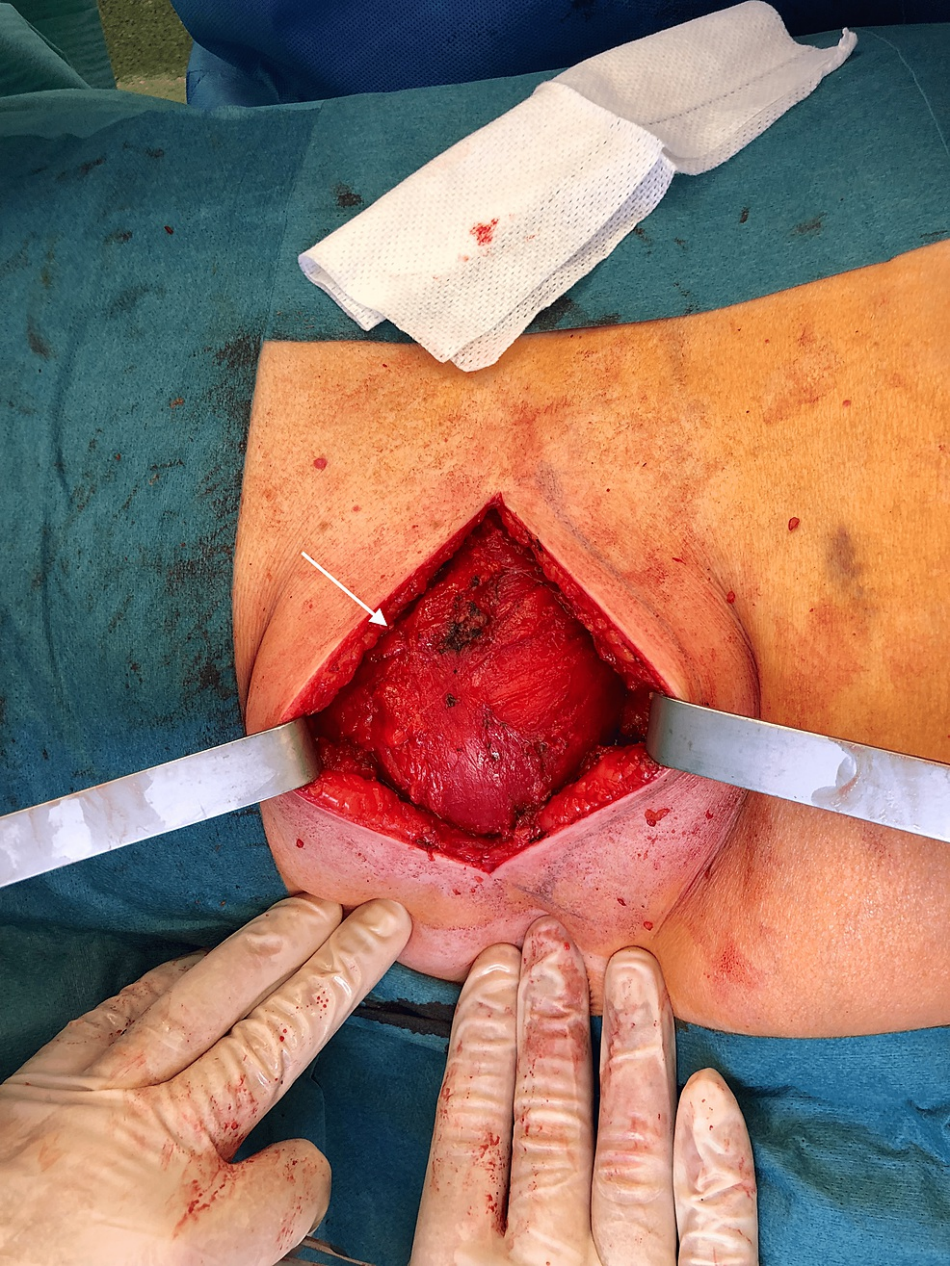
Identification of hernia sac.

**Figure 7:**
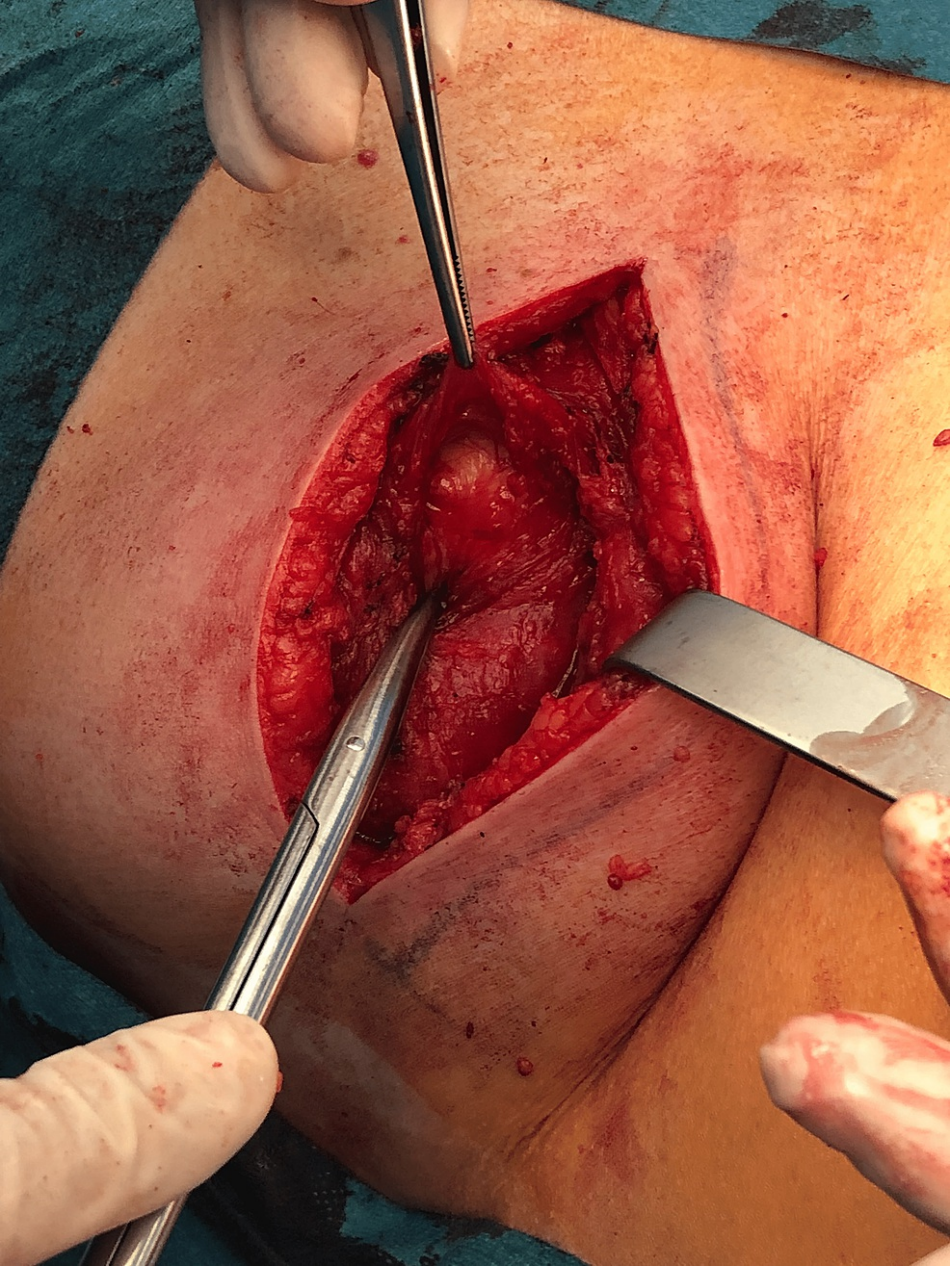
Hernia sac after isolation and liberation of adhesions.

**Figure 8:**
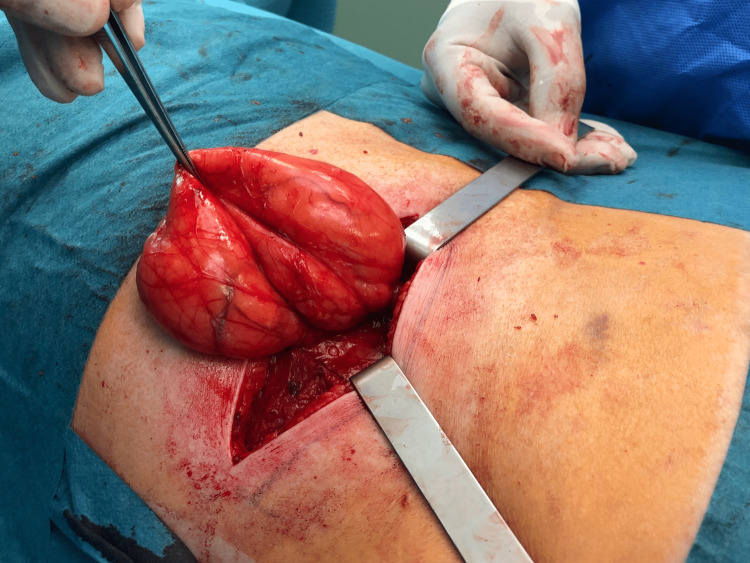
Hernia sac isolated.

After dissection, the hernia sac (15 cm of longest axis) was liberated of its adhesion and isolated (Figures 6-7). Its content was just fat and retroperitoneal vessels (Figure 8).

**Figure 9:**
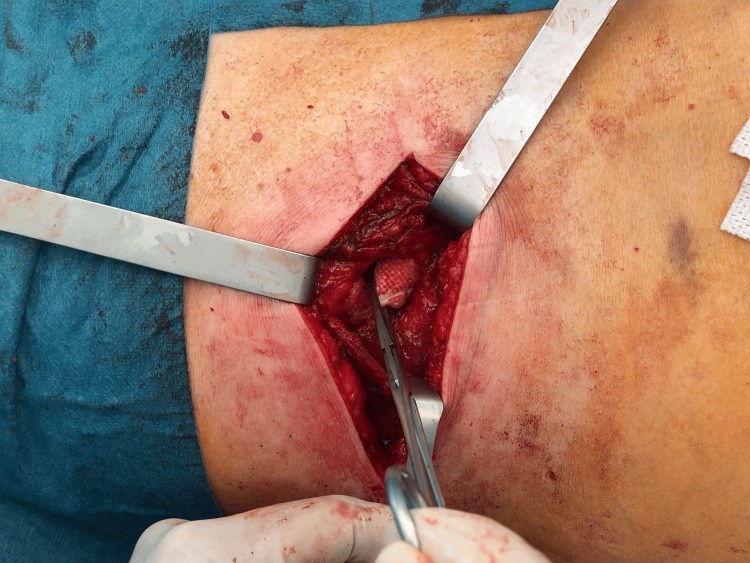
Application of a preperitoneal Ventralex® mesh.

**Figure 10:**
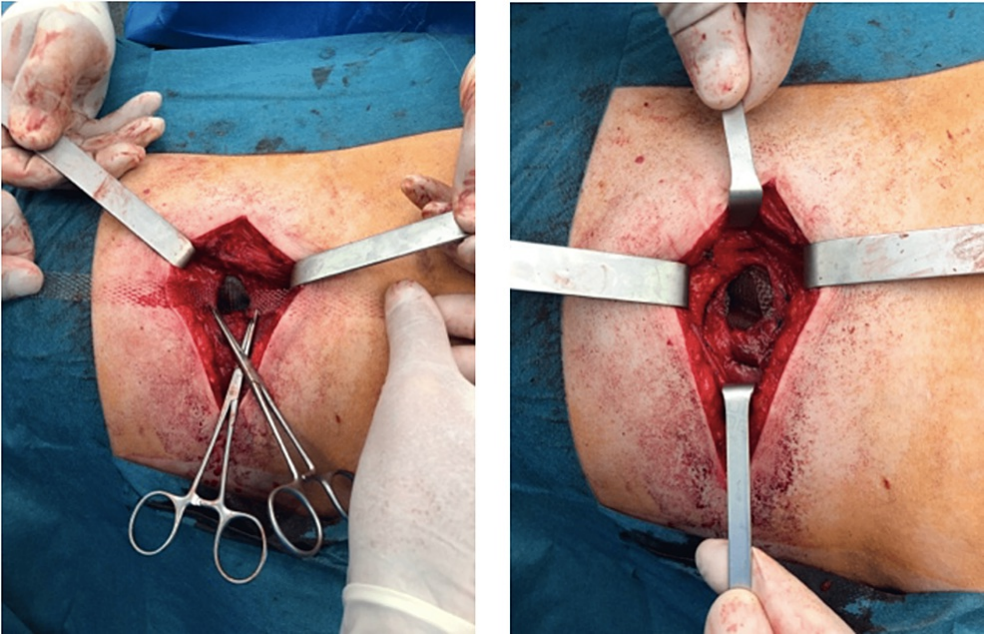
Fixation of preperitoneal Ventralex® mesh with Prolene 2/0.

The hernia content was reduced with no difficulty. The hernia neck measured approximately 5 cm with minor muscular atrophy. A preperitoneal Ventralex® mesh was placed and its fixation was done with separated stitches of Prolene 2/0 (BDI Surgery, Rhode Island)(Figures 9-10).

**Figure 11:**
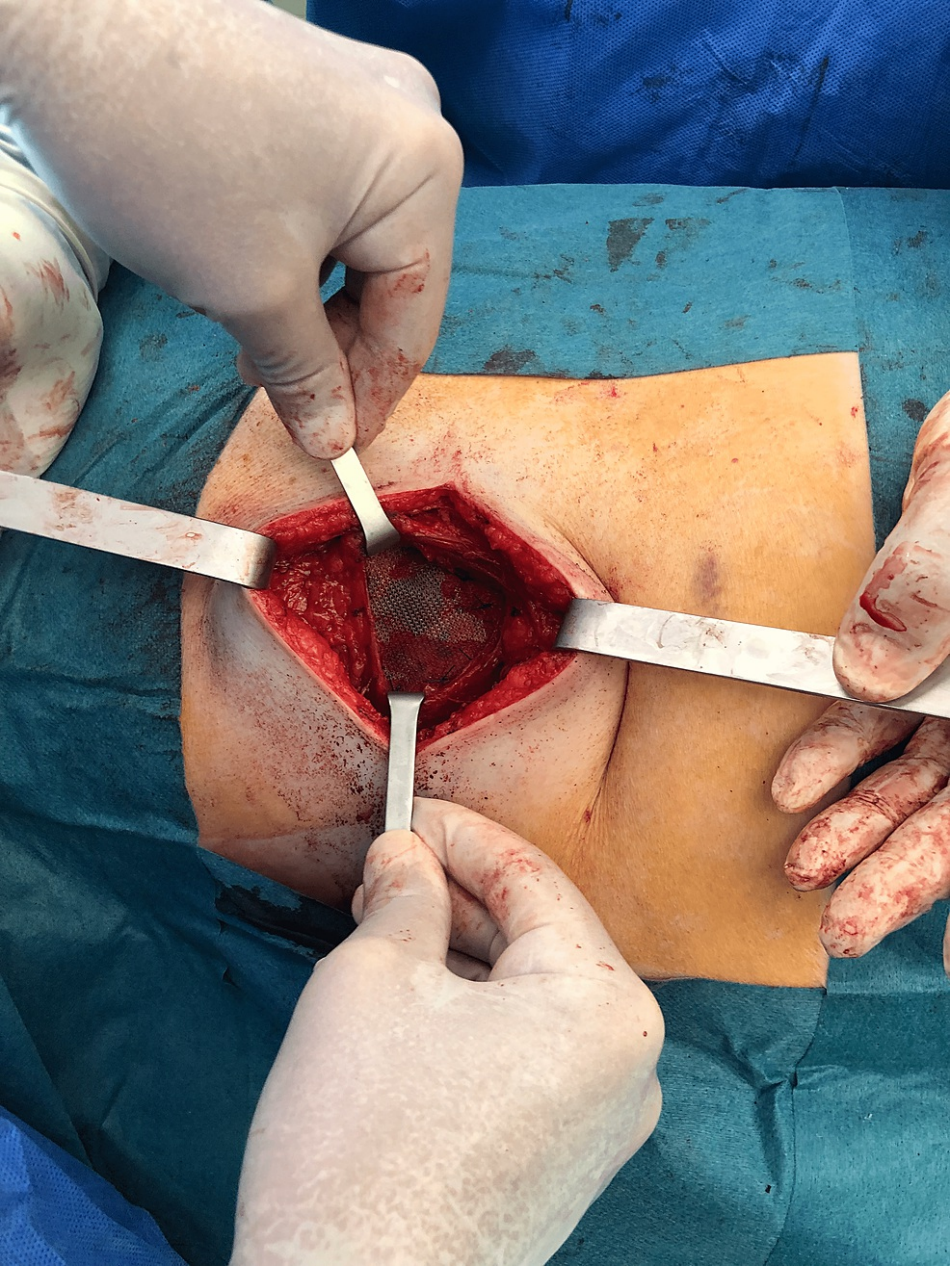
Reinforcement with a subaponeurotic polypropylene mesh fixed with Prolene 2/0.

Reinforcement of the hernioplasty was made with a subaponeurotic polypropylene mesh above the external oblique muscle, fixed with separated stiches of Prolene 2/0 (Figure 11). 

Hemostasis was adequate. A submuscular aspiration drain was placed. Closing was made by plans and staples were used on the skin.

## Discussion

Lumbar hernia is a rare entity, so both diagnosis and treatment can be challenging [2-3]. Clinical examination, conducted in an accurate and scrupulous manner, is still the key tool for diagnosis [2]. The performance of maneuvers to increase abdominal pressure on clinical examination, such as the Valsalva maneuver, is extremely important to avoid misdiagnosis.

Confirmation of this condition can be made by US examination, but it requires a skilled radiologist in order to exclude differential diagnosis and characterize the defect [1]. So, frequently it requires other imaging studies, like CT scan or MRI, for the definitive diagnosis. In our patient, GH was misdiagnosed with lipoma by the US. We needed MRI to clarify the diagnosis and it was helpful for a better description of the muscular walls, the hernia defect, the anatomical relationships, and the size/contents of the hernia. 

The preoperative classification and the experience of the surgeon are important factors in the management of GH and in the surgical approach [5]. The only classification with surgical implications was proposed by Moreno-Egea et al. [7-8] and it divides LH into four types, accordingly six criteria (Table 1). Our patient has a type “A” GH, so the surgical approach could be open with synthetic mesh placement or laparoscopic.

**Table 1:** Classification of lumbar hernias by Moreno-Egea et al. [8].

Characteristics	A	B	C	D (Pseudohernias)
Size (cm)	< 5	5-15	>15	Not applicable
Location	Superior	Inferior	Diffuse	Not applicable
Contents	Extraperitoneal fat	Visceral	Visceral	Not applicable
Etiology	Spontaneous	Incisional	Traumatic	Not applicable
Muscular atrophy	No (minor)	Mild	Severe	Severe
Recurrence	No	Yes (open)	Yes (laparoscopic)	Not applicable
Surgical approach	Open or laparoscopic approach	Intraperitoneal laparoscopy	Open approach	Open approach (double mesh)

The most commonly performed technique is the open approach. It is safe, effective, and less expensive than laparoscopy. Since the first reports, different surgical techniques have been described: primary closure, fascial or gluteal flaps, and the use of meshes [5]. However, these options have proven to be ineffective, probably due to weakness of the surrounding tissues, limited fascial strength, and sewing of the bony portion of the hernia boundaries. In addition, some postoperative complaints are due to hematoma and seroma formation, retraction, compression of nerve terminations, or mesh infection [2].

As an alternative and safe method to the open approach, the laparoscopy approach can be performed. It avoids lumbar incision allowing better esthetic results, improves the visualization of the abdominal wall defect and the relation with anatomical landmarks, allows the placement of a large mesh, and allows faster recovery, reducing postoperative pain and surgical site infection rates [1]. However, some authors suggest that laparoscopy should be preferable for bigger hernias, suspected of visceral strangulation and relapses [4]. Furthermore, experience in the management of abdominal wall defects and advanced laparoscopy skills are needed to perform this approach [5].

The use of meshes and the advent of laparoscopy have by far overcome the previous techniques described, although so far, no method can be recommended as preferable. There are no comparative studies between open techniques and laparoscopy for primitive LH [4]. Treatment choice has to be tailored to a particular patient, including aspects such as defect size, location, content, status of surrounding tissues, and patient affordability [2]. In our patient, we decided on the open approach and the “sandwich technique.” This technique, described in the literature by Sahoo et al. [9] and cited by Ploneda-Valencia et al. [5], is safe, feasible, acceptable, and associated with no short-term recurrence rate. As published in the literature, we showed that “sandwich repair” is a safe procedure with good results. Regarding open approaches to surgery, and especially given the relative rarity of these cases, no procedure has been shown to have definitive advantages over the others [5, 9]. 

## Conclusions

Grynfeltt hernia is rare and requires a careful evaluation for the correct operative management. CT scan or MRI of the abdominal wall can be used for diagnosis and characterization. As a rare hernia, there is no gold-standard surgical treatment. As published in the literature, we showed that “sandwich repair” is a safe procedure with good outcomes.
